# The Way forward for the Origin of Life: Prions and Prion-Like Molecules First Hypothesis

**DOI:** 10.3390/life11090872

**Published:** 2021-08-25

**Authors:** Sohan Jheeta, Elias Chatzitheodoridis, Kevin Devine, Janice Block

**Affiliations:** 1 Network of Researchers on the Chemical Evolution of Life (NoRCEL), Leeds LS7 3RB, UK; 2 Department of Geological Sciences, National Technical University of Athens, 157 80 Athens, Greece; eliasch@metal.ntua.gr; 3 The School of Human Sciences, London Metropolitan University, London N7 8DB, UK; K.Devine@londonmet.ac.uk; 4 Leumit Health Services, Beit Shemesh 9956504, Israel; Jblock@leumit.co.il

**Keywords:** prions, amyloids, origin of life, LUCA, RNA, RNA viruses, chemical relics

## Abstract

In this paper the hypothesis that prions and prion-like molecules could have initiated the chemical evolutionary process which led to the eventual emergence of life is reappraised. The prions first hypothesis is a specific application of the protein-first hypothesis which asserts that protein-based chemical evolution preceded the evolution of genetic encoding processes. This genetics-first hypothesis asserts that an “RNA-world era” came before protein-based chemical evolution and rests on a singular premise that molecules such as RNA, acetyl-CoA, and NAD are relics of a long line of chemical evolutionary processes preceding the Last Universal Common Ancestor (LUCA). Nevertheless, we assert that prions and prion-like molecules may also be relics of chemical evolutionary processes preceding LUCA. To support this assertion is the observation that prions and prion-like molecules are involved in a plethora of activities in contemporary biology in both complex (eukaryotes) and primitive life forms. Furthermore, a literature survey reveals that small RNA virus genomes harbor information about prions (and amyloids). If, as has been presumed by proponents of the genetics-first hypotheses, small viruses were present during an RNA world era and were involved in some of the earliest evolutionary processes, this places prions and prion-like molecules potentially at the heart of the chemical evolutionary process whose eventual outcome was life. We deliberate on the case for prions and prion-like molecules as the frontier molecules at the dawn of evolution of living systems.

## 1. Introduction

Stepping backward from an RNA world into a nucleic acid depleted epoch, we find ourselves in a presumed protein world, i.e., a world that existed prior to the emergence of RNA as one of life’s building blocks. In such a world, what molecules might be expected to be instrumental in the chemical evolution toward living systems? Imagine that we were beginning from this ancient prebiotic protein world, how might we select a molecule capable of driving chemical evolution toward the emergence of life? We might choose one that exhibits durability under harsh conditions, a criterion that has been amply demonstrated for prion proteins (PrPs), which has the tendency to misfold into a rogue isoform. We might also wish to select a molecule that exhibits flexibility, meaning that during times of scarcity, our molecule could be replaced by similar molecules that perform the same functions. Furthermore, given a potential abundance of applications but a limited variety of functional molecules, we might wish to select a molecule that could be used for different purposes at different times. In short, we would aim to select a molecule that exemplifies “thrift”. Prion proteins appear to fulfill the criteria for thrift: they exhibit preservation of function with interchangeability of structures; but they also exhibit diversity of function, despite the same or similar molecular building block structures.

In support of a prion first hypothesis, we infer from theorized “chemical fossils” that suggest life’s origin—molecules such as RNA, NAD, FAD, acetyl-CoA, and ATP—that are presumed to have been around when life first emerged and that have remained largely unchanged until the present day. We argue that prions, too, are chemical fossils that contain important clues within. For example, certain oligopeptides, capable of abiotic formation, are able to mimic properties of contemporary prions, including the ability to self-replicate and to aggregate into diverse structural forms. In addition, prions or “prion-like proteins” are capable of shape-wise self-replication, meaning that they are self-replicating beta sheet conformers, able to transfer steric conformation to progeny molecular entities in non-Mendelian fashion [1,2]. The latter are prion-like proteins which behave like prions (by replication and propagation of neurodegenerative disease), but are not actually prions as exemplified by amyloid-β (Aβ), tau, α-synuclein, and the transactive response DNA-binding protein of 43 kDa (TDP-43). This means prions or prion-like molecules are also capable of “mutation” in the sense that during the process of shape transfer to other “normal and healthy” prions and/or other localized proteins (and depending on the environmental factors), the prion changes its conformation; indicating that prions are capable of a type of mutation [3]. If this definition of mutation is considered to be acceptable then it means that prions did play significant roles in the emergence and evolution of life, as will be demonstrated by taking examples from contemporary biology.

In addition, it is highly probable that at the time of the chemical evolution of life, prions and prion-like proteins afforded protection for newly emerging RNA polymers in the Earth’s early harsh environment. It is during this time-frame that proteins and RNAs forged a link in the form of ribonucleoproteins (RNPs). This link is all too obvious from the fact that ribosomes are RNPs and are indispensable to all cellular life forms, as well as both RNA (and DNA) viruses. In fact, it is the reliance of RNA (and DNA) viruses on the ribosomes for their propagation which highlights the testament to the unique marriage of convenience between protein and RNA during the earliest history of chemical evolution and emergence of life on the Earth.

This paper considers the possibility of prions and prion-like molecules first as a hypothesis for the facilitation of the emergence of life during the early period of the Earth’s history. We begin our investigation with the “clarification of terminologies used” followed by a brief reappraisal of similar hypotheses—primarily those of Chernoff [2] and Maury [4,5]. Next, we introduce the following discussions: the source of prebiotic amino acids; prebiotic peptides; structure of prion proteins; characterization of prions and prion-like proteins; antiquity inventories of prion proteins and amyloids; the adaptive nature of prions; hormesis: reversible binary switch, homeostasis and ion regulation; and RNA amplification and protection. 

PrPs are heritable self-perpetuating protein isoforms which give rise to eventual amyloids. PrPs exist in two forms, namely, the normal healthy form, PrP^C^ (C = cellular isoform), and the infectious form, PrP^Sc^, where Sc = from scrapie, a neurodegenerative disease in sheep and is a misfolded isoform of the PrP^C^. The latter, as above, contains small segments of prion domains (PrDs), typically to be found at the N- or C-terminus, which initiate the propagation of PrP^Sc^ by acting as a substrate and subsequently growing into an aggregate of abnormally folded PrP^Sc^. The synthesis of abnormally formed PrP^Sc^ then acts as a template for the next generation of complete infectious isoform aggregates; noting that when a prion converts a normal protein into another prion, the structure is conserved. Finally, prion-like proteins are normal cellular proteins which behave as potential prions, e.g., amyloid-β (Aβ). Compared to PrPs, the amyloids which they “spawn” are much larger microscopic structures containing mostly β-sheets rich (and α–helices poor) and they came later in the evolutionary time-frame [2]. Thus, the stance taken by the authors is that prions may have been instrumental in the emergence of life on Earth.

## 2. A Brief Reappraisal of Similar Hypotheses

The work of Maury [4,5,6] on amyloids as a foundation for the origin of life will not be appraised here, as the authors believe that much of the basis for the origin of life resides in prions and prion-like molecules, as promulgated by [1,2,3] during much earlier publications. Although Maury’s work is principally concerned with amyloids, we note that in his later papers he does bring prions into the equation (e.g., [6]). The main theme of this manuscript, however, is that prions and prion-like molecules are predominantly priori, as opposed to amyloids which are the product of the aggregation of PrPs resulting in the formation of misfolded aggregates such as fibrils, scaffolds, and nanotubes. Such misfolded β-sheet structures, although they have been shown to be both versatile self-replicators and catalytic, are large enough to be easily observed by 2-dimensional X-ray diffraction and thus we question the veracity of these huge structures arising de novo. We adhere to the idea that oligomer sized prions were the initiators of the process of the emergence of life, as will be demonstrated later in the manuscript. This manuscript develops the ideas of Chernoff, who first published the possible involvement of prions and the possibility of Lamarckian evolution during the emergence of life [1,2]. While we shall confine our discussion largely to prions and prion-like molecules, however, where necessary, amyloids will be brought into focus.

## 3. Source of Prebiotic Amino Acids

One of the major distinguishing features of proteins is that the monomers from which they are constructed (amino acids) are held together by peptide bonds (R-CONH-R′). Amino acid monomers are necessary for the construction of all manner of peptides—the workhorse of cellular biology. While the original source for the first protein monomers is unclear, one possibility is that they were formed within the Earth’s atmosphere. This possibility was demonstrated empirically by Miller in 1953 [7], who subjected highly reducing gas mixtures of dihydrogen (H_2_), ammonia (NH_3_), methane (CH_4_), and water vapor to a high electrical discharge, simulating the effects of highly charged atmospheric electric lightning, the type that occurs in volcanic plumes rather than that which occurs over desert landscapes [8]. Miller’s experiments demonstrated that both proteinaceous biologically relevant and non-biological amino acids could be formed in this manner, including glycine, alanine, aspartic acid, valine, glutamic acid, and phenylalanine [7,9]—see Table 1. A total of eighteen amino acids were made under Miller’s experimental conditions and the comprehensive list of amino acids was identified using sophisticated HPLC/GCMS instruments on resins contained in the vials dating back to 1953, which were found in Professor Miller’s office after his death in 2007.

Alternatively, the first proteinaceous α-amino acids might have been formed at the head of impactors during periods of intense bombardment of the early Earth, between 4.3 to 4.0 billion years ago; within atmospheric lightning of the volcanic plumes [7,8,9,19]; within alkaline hydrothermal vents [20]; and at the interface between dense and less dense layers, for example, in the heavy, orange hazy atmosphere of Saturn’s moon, Titan [21]. The largest amounts of organic compounds, including the first amino acids, arrived on the Earth by way of carbonaceous chondrites and similar impactors. Carbonaceous chondrites such as the Aguas Zarcas meteorite, discovered in Costa Rica [22], and the Murchison meteorite, found in Australia, are postulated to date back to the time of the early Solar System and have been shown to contain numerous different amino acids, including other biologically important organics (e.g., adenine, guanine, uracil). These amino acids, as well as other organic molecules would have been delivered onto the surface of the Earth by carbonaceous chondritic meteorites during the Heavy Bombardment period [23,24,25,26]. Testimony to the Heavy Bombardment epoch are the craters on the Moon which have remained pristine to date, in the absence of those weathering processes experienced on Earth.

## 4. Prebiotic Peptides

Once amino acids made their appearance on the early Earth, the next step was that the synthesis of chains of various lengths of prebiotic peptides could commence. Iqubal et al. [27] demonstrated that dipeptides, for example, could be made on heterogeneous, metal ferrite (e.g., nickel ferrite, NiFe_2_O_4_), nano-particle surfaces. Ikehara [12,28] also demonstrated the possibility of making oligopeptide chains using repeated dry/heat cycles. Similarly, Ferris et al. [29] showed that lengths of 55 amino acid chains could be formed on the surface of clays such as illite and hydroxylapatite; other clays used in such experiments include kaolinite and montmorillonite, signifying that clays are, by and large, a realistic possibility. The essence of such experiments being that peptides could be synthesized abiotically. Given such abiotic experiments, Greenwald [30] further showed that amino acids could be condensed into amyloid aggregates. Taking all these observations into consideration, it is not beyond the realms of probability that a peptide world was a realistic possibility, noting that prions are, in essence, peptides.

**Figure 1:**
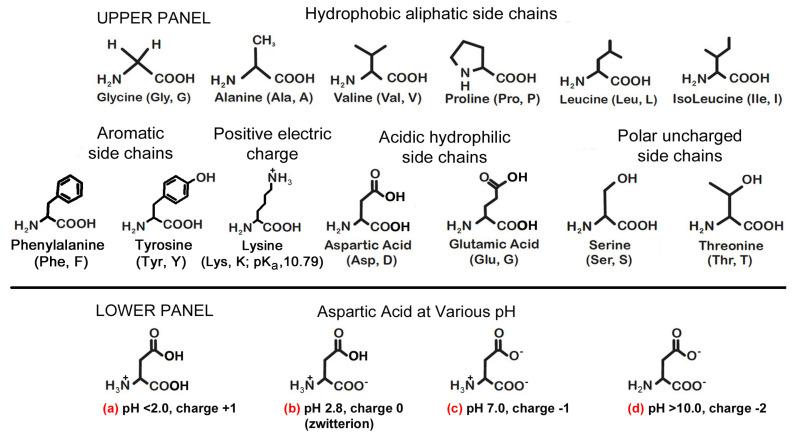
**Upper panel:** shows thirteen amino acids that were thought to be present during the chemical evolution of life as reported in Table 1 column (g) [14]. **Lower panel**: shows aspartic acid has net protonated amide ($NH_{2}H^{+}$) side chains in (**a**–**c**) at various pH including a zwitterion (**b**) and a zero charge at pH 2.8. It has been reported that both aspartic acid and glutamic acid are particularly relevant in 65% of catalyst residues because of their ionic charges, remembering that it is the interplay of electrons which brings about reactions—*cf* the three hydrophobic amino acids used in Ikehara’s experiment Gly > Ala > Val. Likewise, serine and threonine (with their polar, uncharged, side chains), and tyrosine are also important in 27% of catalyst residues. To complete the comparison, hydrophobic amino acids (Gly, Ala, Val, Pro, Leu, and Ile) feature only 8% of the time at the active sites of enzymes [16,18]. In (**d**) aspartic acid represents as having overall charge of −2 when the pH exceeds 10.0.

Furthermore, expanding on Ikehara’s important empirical evidence for a “[GADV] protein world [protein-first] hypothesis”, he was able to make chains of oligopeptides from prebiotic glycine (G), alanine (A), aspartic acid (D), and valine (V) [12,28]. Subsequently, according to both Bartlett (2002) and Georgiou (2018), of the four amino acids deployed by Ikehara, aspartic acid with its various protonated states (Figure 1 lower panel) is particularly relevant as far as catalysis, in general, is concerned: Asp (2) > Gly > (4) > Ala (4) > Val (4); the numbers in brackets are an indication of genetic code redundancy, as compared to biogenic amino acids which have a redundancy of between 1 and 2, for example methenamine and cysteine respectively [16,18]. Ikehara further demonstrated that these oligopeptides could act as catalysts for the hydrolysis of peptide bonds of bovine serum albumin, noting that he does not report examples of anabolic activities. Additional examples of catalytic triads of amino acids (Table 3) are taken from Georgiou’s paper [18], which are groups of three vital amino acids essential for catalytic activities found at the biologically active sites within the enzymes. The catalytic triads made only from initial available meteoritic α-amino acids (rows 1, 2, and 3) in general are essential because they act as catalytic unit sites within enzymes, especially those which would have been active during the prebiotic chemical evolution epoch. These initial α-amino acids were not so “chemically complex” because of their high levels of hydrophobicity (Gly, Ala, Val, Pro, Leu, Ile, and Phe) compared to biogenic amino acids, namely His and Cys with their imidazole rings and thiol groups. The triads made from His and Cys (rows 4 and 5, respectively) display the highest catalytic activities propensities [3,16,18].

Nevertheless, revolutionary experiments, such as Ikehara’s, do demonstrate that oligopeptides made from the reported top 10 α-amino acids listed in Table 1, column (e) could act as catalysts (albeit catabolic reactions), one assertion being that if peptides can carry out “any” enzymatic reactions, then they must also be able to carry out forward anabolic reactions. Assuming anabolism is possible, which is highly probable, then during the early stages of the chemical evolution of life, such enzymes would not need to be 100% efficient; peptides with mild catalytic activities could have sufficed [31]. Such chemical reactions would have been edging towards emergence of life.

## 5. Structure of Prion Proteins

**Figure 2:**
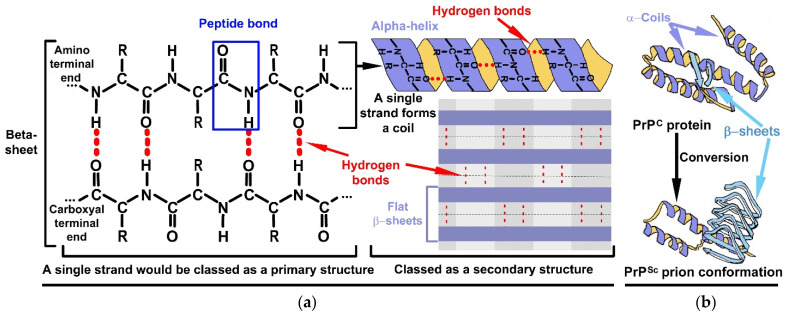
(**a**) Formation of secondary level structures in the form of α-helices and β-sheets. The red dotted lines are an indication of the presence of hydrogen bonds between two peptide strands. The amino acids: glycine, alanine, aspartate and proline are widely distributed in α-helices; valine, isoleucine and tryptophan are commonly found in β-sheets [3]. Noting that tryptophan is not included in the top thirteen prebiotic amino acids indicated in Table 1. This raises two possibilities. Firstly, that initial proteins were much “simpler”, being made from only the thirteen amino acids. Secondly, that tryptophan is absent in the chemical inventory of comets [14] and was, thus, acquired later during the chemical evolution, meaning that they are probably biogenic in nature [18]. (**b**) within the context of PrPs, the PrP^Sc^s precipitate out into tightly packed tertiary structures which means they are able to withstand harsh environmental conditions when compared to RNA shapes. This figure also depicts that PrP^C^ is more α-helices orientated than β-sheets. This situation is reversed in PrP^Sc^.

Normal functional contemporary PrPs are exclusively made up of amino acids and thus are therefore proteinaceous. These protein structures exhibit unique properties in that they are very compactly packed structures, as in Figure 2b which suggest that there is a possibility that PrPs played an instrumental role in the chemical evolutionary processes leading to the emergence of life on Earth, e.g., affording protection, as in Section 10**,** to newly emerging RNAs as well as forging relationships with each other to form ribonucleoproteins. To understand the nature of PrPs, it is first necessary to examine the structure of a protein in general. What are proteins? By definition, all peptides are composed of a selection of 20 different biologically important α-amino acids (Table 1) that have been ordered precisely, as specified by mRNA in biology, also noting that they can be made abiotically, for example on clay surfaces [27,29].

Proteins are categorized into four structural levels; likewise, prions (the theme of this paper) can also attain four levels of structure. However, the initial prions would have been made from a selection of 13 prebiotic α-amino acids, as depicted in Figure 1 upper panel and Table 1.

When amino acids are strung together by peptide bonds, similar to beads on a string, the structure formed is said to be a primary structure—it is simply a single straight strand of amino acids. A good example of primary level conformations, in the context of this paper, would be a biologically active prion domain (PrD). Such domains are generally oligopeptide motifs, which are embedded within the larger PrP strands [32,33]. Then, secondary structures are formed when amino acids within prion chains interact with one another via the formation of hydrogen bonds, as identified in red in Figure 2a. 

The two types of secondary structure observed in PrPs are α-helices (coiled ribbons) and β-sheets which are flat (Figure 2a); these sheets could be parallel or antiparallel. Although the core of the proteins contains hydrogen bonds, with the essence of these bonds being that they confer certain levels of rigidity and compactness within proteins. However, proteins also contain disulfide bridges in general which are formed due to the presence of the sulfur containing amino acid, cysteine (with its thiol, S-H, side chain) which is conspicuously absent in the organic inventory of meteorites [14]. Cysteine is thought to be of biogenic origin (i.e., made by living entities) and in terms of its relative importance, ranks almost at the bottom according to Trifonov (column a, Table 1), meaning that it may have been acquired later on during the evolutionary time frame [10,18]. The implication of the absence of disulfides bridges implies that PrDs were somewhat simpler when compared to, for example, proteins which contain cysteine.

The PrPs’ complex shapes are a result of formation of tertiary structure from interaction between the secondary shapes pertaining to α-helices and β-sheets, as depicted in Figure 2b. The tertiary aspect attained depends on a number of factors as follows: firstly, on ionic interactions due to the presence of ions of sodium (Na^+^), for example; noting that ionic interactions are found deeply embedded within the tertiary and quaternary shapes. Such ions interact with negatively charged carboxylic groups ($R-COO^{-})$ on the amino acids, forming more resilient ionic bonds—for example, at various pH, the acidic side chains on aspartic (and glutamic acids) becomes deprotonated in particular (c) and (d) in the lower panel of Figure 1, thus attaining negatively charged states. The strong ionic bonds make a PrP attain a shape which is a tight and compact conformation which in turn confers resilience, a tertiary structural feature of the PrP^Sc^ (Figure 2b). Secondly, the presence of these ionic charge forming amino acids gives dissolving properties during folding of PrPs. Thus, such amino acids, at pH 7.00 for instance, would generally orientate towards the “outer” edges as they interact with the bipolar water molecules, thus aiding the folding of PrPs. Thirdly, the relevance of the location and number of amino acids with hydrophobic aliphatic side chains, for example, glycine, alanine, valine and leucine [34], as their side chains more often than not point “inwards” away from bipolar aqueous solvents (e.g., water), and thus they are almost always located deep within PrP structures. Fourthly, the presence of another aliphatic hydrophobic and nonpolar amino acid, namely, proline (technically cyclic and an imino acid), within the PrP chains adds an additional dimension; cyclic proline, unlike the remaining proteogenic amino acids, is denied the opportunity of free rotation on either side of its peptide bond. This conformational rigidity stabilizes the secondary structure of the protein chain by comparison with other peptide residues. Another important aspect of proline is that it does not carry an additional amide hydrogen atom so cannot be a hydrogen bond donor, but the proline nitrogen can act as a hydrogen acceptor. Finally, the shape (e.g., β-sheets, α-helices, and helix twist lengths) is also determined by the solvents that PrPs are present within, meaning that they may assume one conformation when in an aqueous solution and a different conformation within oil and aqueous/alcohol mixtures. The space is absolutely germane because the majority of the enzymatic activities take place within cell membranes as well as cytoplasm. When presented with α-helices and β-sheets made from the thirteen top prebiotic amino acids and in the presence of various shape conformation conditions listed above, it is possible to arrive at tertiary structures for PrPs, especially in the presence of deeply imbedded ionic bonds. This is despite the absence of disulfide bonds. Finally, a quaternary structure refers to “meta”-structures such as fibrils, scaffolds, and tubes in which the underlying building blocks are principally secondary and tertiary protein conformations. The meta-structures are largely a feature of amyloids, which is the basis for Maury’s hypothesis for the emergence of life.

## 6. Characterization of Prions and Prion Proteins

Prions are best described in the context of human-centric neurodegenerative diseases such as Kuru and Creutzfeldt-Jakob, as well as Alzheimer and Parkinson diseases [35], which are principally thought to be caused mainly by rogue proteins of specific natures; what is remarkable is that PrPs are encoded in the host’s genome [36]. The healthy cellular (C) version of a PrP has many regulatory functions in human biochemistry and physiology (e.g., synaptic vesicle trafficking) and is termed PrP^C^. The toxic and disease-causing isoform of any PrP^C^ is designated as PrP^Sc^. In terms of structural difference between the two: the former contains more α-coiled configurations when compared to β-sheets; the latter is a misfolded isoform consisting of enriched β-sheets (relative to α-coils), which tend to be much less soluble and with a propensity to aggregate compared to their α-helical forms (Figure 2b). In essence, what happens is that a PrP^C^ can misfold to generate a PrP^Sc^, which then has a completely different shape compared to the healthy PrP^C^. This isoform version then self-propagates further by template-directed conversion of healthy PrP^C^s and misfolding of other normal proteins. It is believed that the resultant misfolded shapes are the diseased agents which are being transmitted [37].

Typically, a PrP may be large, but the PrD itself may be quite short. For example, in one large bacterial prion protein derived from *Clostridium botulinum*, the prion motif residing at the 92–112 segment is only 21 amino acids long [33]. Such prion motifs are capable of self-propagation even when separated from the rest of the protein moieties that contain them [38].

Both PrPs and prion-like proteins themselves have the ability to act as templates for transmission of their shapes onto other proteins. For example, in the yeast *Saccharomyces cerevisiae*, when the protein Rnq1p is aggregated in its [PIN+] prion form, it promotes the de novo appearance of the prion form [PSI+] in the *S. cerevisiae* protein Sup35 [39]. Furthermore, thanks to the actions of hydrophobic prion or prion-like side groups, these amyloid-forming proteins can also transmute their folded-conformational shapes onto normal dominant variants of the same protein types [40]. This ability of prions and PrPs to effect shape change (i.e., attribute-change) in nearby molecules may be seen as the transference of phenotypic shapes, effectively a type of Lamarckian evolution. Another example being that when at the dawn of life’s emergence, an entity gains a nucleotide(s) or “protogene” due to horizontal gene transfer it could be construed to be non-Darwinian inheritance—i.e., Lamarckian evolution. Further, the required threshold for Darwinian evolution to become a viable proposition is uncertain, which has more to do with the passage of genotypes [1,2,3]. The comparison between Lamarckian and Darwinian evolution is necessary because the former is rapid and occurs more or less instantaneously, as phenotypic shapes are passed on intact, whereas the latter is a slowly occurring process over longer durations. Thus, Lamarckian evolution would have been exceptionally important in a prion-first world, during the era of a scarcity of chemical informational molecules such as RNAs. The reader may wish to further consult Chernoff’s paper: “Mutation processes at the protein level: is Lamarck back?” [1,2,3], which expounds on Lamarckian evolution.

Alternatively, since prions and PrPs can form amyloid aggregates spontaneously in the absence of prion-induced transmission [41], another way of looking at this process of phenotypic transmission would be to see it as an evolutionary process that occurs in a non-Mendelian fashion, ad hoc and/or de novo [1]. (Recall: Mendelian traits are expressed in certain ratios). Indeed, given that this shape transformation can occur spontaneously, as well as by being initiated by PrDs seeds, this would denote an evolutionary “behaviour” by prions which is due to Lamarckian evolution. It is now becoming clear that even Darwinian evolution may also have a hand in the shape transformation as reported by Li [42].

Prions, being compact and infectious agents, are remarkably resistant to extreme environmental conditions such as high temperatures (up to 134 °C for 18 min), i.e., simmering heat from volcanoes and impactors, and heat generated by radiogenic elements from within the Earth’s surface (e.g., ^40^K, ^235^U, ^238^U and ^232^Th); particle radiation (e.g., H, H^+^, D, D^+^, He, He^+^, and He^2+^); UV light from the Sun and cosmic rays, as well as strong acids (due to acidic rainfall) and formaldehyde treatments, in fact, any likely conditions that would affect the nucleic acids; that is, the environmental conditions that existed on the early Earth 4.3–4.0 billion years ago. Thus, prions and PrPs stand out as protein-based molecules of unique “toughness”—tough enough to survive even the harsh conditions of the early Earth [43,44,45,46].

## 7. Antiquity Inventories of Prion Proteins and Amyloids

**Figure 3:**
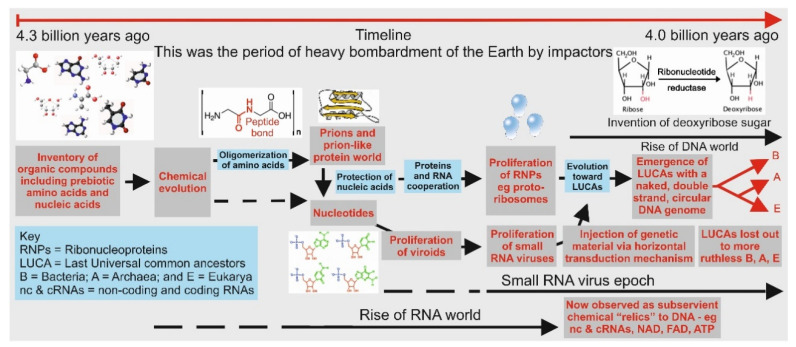
Pictorial representation of major events during the emergence of the three domains of life, namely, Archaea, Bacteria, and Eukarya. It is highly probable that both Archaea and Bacteria emerged from two resilient LUCAs based on their cell membrane compositions and biochemistries (see [50], with Eukarya being a chimera of the former two domains.) It is believed that viruses had an independent origin, probably emerging during the RNA world era.

PrPs (and amyloids) are ubiquitous and thus are commonly represented in all three domains of life, as well as amongst both DNA and RNA viruses with their coding regions for PrPs and amyloids [47,48]. Domains of Archaea and Bacteria are presumed to have emerged directly from the resilient Last Universal Common Ancestors (LUCAs) [49] approximately 4.1 billion years ago [50]. Figure 3 represents a timeline for the emergence of the three domains of life including signposts of major events during the evolution of a protein world, noting that this was also the period when the Earth’s surface was heavily bombarded by impactors. It is to be noted in Figure 3 that viroids were probably the first to emerge and were most likely present during the earliest chemical evolution of life as indicated in Figure 3. They are pathogenic agents and are found to be tiny (246 to 463 nucleotides long) single-stranded circular RNA molecules that are “geneless”. That is to say they do not code for triplets for amino acids; further, they do not contain any coded genetical information [51,52]. What viroids have is structural information and it is this which is thought make them pathogenic agents. They propagated by template directed replication mediated by rudimentary prions and/or enzymes [12,28].

Despite the fact that Archaea and Bacteria exhibit all of the defining characteristics of cells including the encoding of genomic information into DNA, mature ribosomes, and sophisticated biomembranes [53,54], they are distinctly different from one another so as to be classed as two distinct domains [50,55,56]. Nevertheless, species from both of these domains have been found to exhibit coding regions for PrPs. Eukarya, the third domain, presumed to represent a chimera of Bacteria and Archaea, also emerged with an inventory of PrPs. Thus, it is likely that two vastly different types of resilient LUCAs harbored the PrPs encoding regions. How did the PrPs come to be part and parcel of both the Archaea and Bacteria domains? The answer may be that these encoding regions were introduced by viruses—in particular, by the small RNA viruses such as retroviruses (e.g., human hepatitis D virus with its genome size of 1.7 kilobase), since it was highly probable that these small viruses emerged during the RNA world era, as noted in Figure 3.

## 8. The Adaptive Nature of Prions

Some of the most extensively studied prions and PrPs are those of the yeast *S. cerevisiae*, including the prion-forms [PIN+] for the protein Rnq1p; [URE3] for the protein Ure2p; and [PSI+] for the protein Sup35. These yeast proteins demonstrate the remarkable flexibility inherent to some prions. When the yeast *S. cerevisiae* PrD in Sup35 is substituted either with the PrD of *Cb-Rho* (prion protein from the bacteria *C. botulinum*) or with the PrD of LEF-10 (DNA baculoviruses prion protein), the [PSI+] prion forming behavior of Sup35 is retained [48,57]. In addition, the number of species within RNA Picornavirales order is only 31.88%, yet it has the second highest p-value of 0.7579 when it comes to identification of PrDs in viral proteomes; compare this to the number of species within the DNA Herpesvirales which has the highest percentage of 71.80, yet the lowest p-value of <0.0001. The essence being that PrDs proteomes are highly expressed in Picornavirales [47]. Similarly, in [PIN−] strains of *S. cerevisiae,* the yeast prion [URE3] and the artificial prion [NU+] are capable of substituting for [PIN], thus preserving the prion-forming behavior of [PIN]. Such interchangeability of PrD’s could have performed a critical adaptive role during times of scarcity [32,58]. Furthermore, one PrD may substitute for another, and also the fact that similar PrPs are capable of serving as building blocks for diverse morphological forms and functions stands in contrast to the relative inflexibility of chemical informational molecules, such as RNA. RNAs do not possess this degree of flexibility of structures and cannot by themselves bring about interdependent networks of metabolic processes [59]. To the extent that an evolved function expresses an environmental need, this observation suggests that prions and PrPs probably arose marginally prior to RNA informational encoding, in a world of greater scarcity of resources. Prions and PrPs may thus be said to belong to a pre-RNA world in which the building blocks of life were simpler and scarcer. 

## 9. Hormesis: Reversible Binary Switch, Homeostasis, and Ion Regulation

Many prions and PrPs exhibit another interesting and exceedingly simple behavioral phenomenon: hormesis. If it is presumed that prions and PrPs are ancient, then hormesis is likely to be ancient as well. Indeed, as will be seen, hormesis may have played a key role in driving evolving chemical forms toward life’s emergence by means of homeostasis.

Hormesis is a simple reversible binary switch phenomenon characterized by a U-shaped dose response curve. According to the hormetic pattern, levels of exposure to an agent within the central portion of the curve exert an optimizing or multiplicative effect, whereas levels of exposure at the ends of the curve exert a damaging or reductive effect. For example, in bovine spongiform encephalopathy, the infectious prions respond to Congo red according to a U-shaped hormetic curve: thus, 1µM Congo red increases the formation of prions by directly effecting the PrP conformation, whereas 100µM Congo red maintains the non-prion conformation of PrP, while simultaneously suppressing the prion conformations [60].

**Figure 4:**
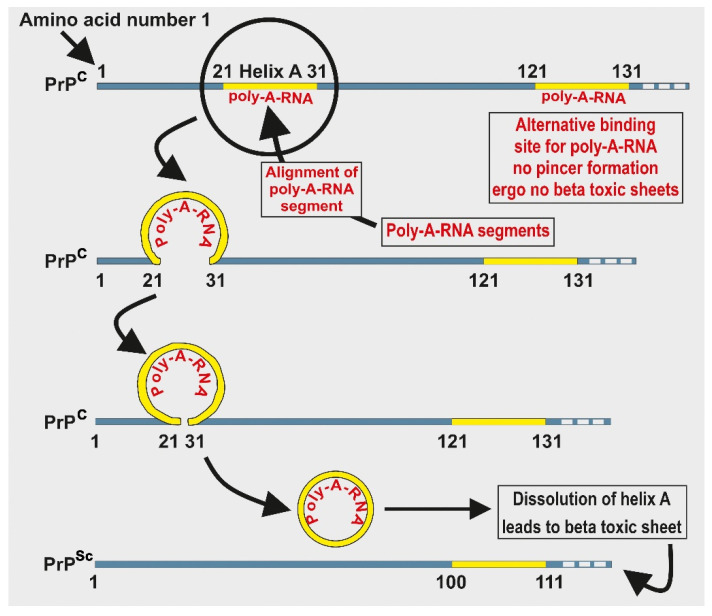
Depicts the formation of a pincer when poly-A-RNA attaches at the 21–31 amino acid segment on the PrP^c^. The pincer is eventually removed and dissolved. The figure also illustrates an earliest possible example of interaction between peptides and nucleic acids.

If, as the evidence suggests, prions were present during the prebiotic chemical evolution of life, then it is reasonable to presuppose that such primitive molecules like prions displayed hormetic behavior, prior even to the emergence of a LUCA. To demonstrate the application of hormetic behavior of prions, consider the following example: a primitive but potentially reversible binary switch is illustrated by the infectious prion in scrapie, PrP^Sc^. If a polyadenosine RNA (poly-A-RNA) fragment binds to PrP amino acid segment at 21–31 of helix A, a pincer-like structure forms between helix A and the polybasic domain, encapsulating the RNA fragment as in Figure 4. This pincer-like structure is necessary for dissolution of helix A on PrP^Sc^ and it is required for the formation of toxic beta sheets. If, on the other hand, a poly-A-RNA fragment binds to the PrP at segment 121–131, steric clashes prevent formation of the pincer-like structure, thus preventing formation of toxic beta sheets [61]. This primitive symbiotic relationship between a single PrP and one or more RNA molecules illustrates how the earliest symbiosis between prions and RNA molecules might have developed.

The PrP model has been used to illustrate the extent to which hormetic self-regulation might be simplified—the model involves only a prion and its ligand, and the prion’s potential self-regulation by steric forces is so simple as to be conceivable in a prebiotic protein world. This process demonstrates one possible means by which the earliest homeostasis could have occurred during a chemical evolutionary phase, preceding the emergence of LUCA. If indeed the survival drive began with a prebiotic regulatory switch similar to the above, what might the switch have been regulating? One possibility is that primitive systems were regulating heavy metal ions such as copper (Cu^2+^), iron (Fe^2+^ and Fe^3+^), and zinc (Zn^2+^) [17]. These metallic ions played very important catalytic roles during the early biological systems as they are also potent oxidizers (e.g., [27]). Without regulation and organization, the influence of such metal ions could have been highly destructive to the emerging life forms. 

## 10. RNA Amplification and Protection

Although oligomers of both nucleotides and amino acids, namely, RNA and peptides, respectively, can be made relatively easily on clay surfaces [29,62], their survivability in the ozone lacking harsh environment on the early Earth is a different question; RNAs are rapidly destroyed especially if they are present in a watery environment [63,64]. Proponents of the RNA world hypothesis argue that RNA oligomers are “protected” within niche places, such as rock crevices (e.g., [65]), or in the small clay bubbles of alkaline hydrothermal vents [20,66,67] and could have escaped degradation caused by environmental factors. Current models for the RNA world posit that the presence of RNA in open, watery environments was an unlikely scenario [68,69,70] and from this presupposition, it may be deduced that the prebiotic world must have been one with a scarcity of nucleotides. 

There are also other challenges to the RNA world hypothesis. Even given a plentiful supply of monomers and a vigorous abiotic synthesis of RNA oligomers, there remains the question as to how such oligomers might have been amplified; remembering that the rate of oligomerization needs to exceed the rate of destruction. The required rapid amplification of RNA oligomers may need the presence of an RNA replicase enzyme for template-directed complementary RNA replication, in order to increase the much needed rapid amplification rate [55]. If, indeed, RNA amplification depends on protein catalysis and cannot proceed without it, then it must be concluded that prebiotic protein must have arisen marginally prior to rise of RNA—i.e., a protein-first hypothesis, though in this case, we are promulgating prions and prion-like molecules first hypothesis [71].

How can an excess of RNA be achieved? The answer must reside with proteins (specifically peptides). In 2012 Maury showed that the initial self-replicator (which can increase its own copy number but not replicate RNA molecules) was made from six prebiotic amino acids (Table 1), namely, glutamic acid, glycine, serine, valine, alanine, aspartic acid, with the following sequence: Glu-Gly-Gly-Ser-Val-Val-Ala-Ala-Asp [11]. Furthermore, it may be recalled that PrDs are small lengths of oligopeptides [32], e.g., an oligopeptide of only seven residues, Gly-Arg-Arg-Gln-Gln-Arg-Tyr, has been isolated in yeast prion Sup35. Further, if we extrapolate that PrDs of between 7 and 21 amino acids lengths are routinely found in living entities and also note that the PrD of *C. botulinum* is only 21 amino acids long and made up of only three types of amino acids, namely, Ser, Asn, and Phe: NNNNSNFNNNSNNNSSFNNSN [72], such PrDs could have played a role during chemical evolution. (Although Asn is not in the top thirteen list of prebiotic amino acids). The PrD’s roles would include aiding in the polymerization and elongation of other proteins, allowing for the generation of longer chains which were resistant to hydrolysis [73]. The foldimer computational model hypothesis [74] posits that these hydrophobic chains initiate and induce protein folding [34] (Figure 2b), noting that the three pairs of amino acids: glycine, valine, and alanine, in Maury’s initial replicator have hydrophobic side chains [11]. The hypothesis also suggests that folding is initiated by hydrophobic domains within protein molecules and confers compact structures and resistance to ever fluctuating environmental stressors of the time [75].

PrP structures, by and large, are far hardier when compared to RNA in the face of harsh environmental conditions. As a consequence, in contemporary biological systems, prions are often deployed in a nucleotide/nucleic acid-protective and sequestering role; for the chaperoning, sequestration, or uptake of nucleic acids. For example, when the *S. Cerevisiae* protein Ure2 assumes its non-prion conformation, it regulates nitrogen metabolism and interferes with uptake of ureidosuccinate (USA), an intermediate in uracil biosynthesis. However, when Ure2 assumes its prion formation [URE3], it enables uptake of USA, with consequent augmentation of uracil manufacture [33]. Similarly, the human prion proteins TDP-43 and FUS (proteins associated with amyotrophic lateral sclerosis and frontotemporal lobar degeneration) play a role in sequestration and alteration of RNA. The prion-like FUS protein actively sequesters RNA binding proteins such as hnRNP A1 and hnRNP A2; and a 35 amino acid fragment of the prion-like protein TDP-43 (TDP-35) binds to RNA and triggers formation of cytoplasmic inclusions that alter RNA processing [75,76].

The Ure2 example described above is particularly enlightening, in that it shows how a single prion protein can support more than one foundational life process. When in its non-prion conformation, Ure2’s support for nitrogen metabolism illustrates how a prion-protein can be positioned at the center of the foundational metabolic processes referred to in the metabolism first hypothesis. (This hypothesis promulgates that the network of pathways, cycles, hypercycles emerged first as opposed to genetic first.) When in its prion conformation, Ure2’s [URE3]’s support for uptake of nucleic acid building blocks illustrates how PrP can be positioned at the center of the foundational metabolic processes referred to in the RNA world hypothesis—this is an example of genetic first hypothesis.

PrPs also interact with nucleoproteins via stress granules, where coding mRNAs and non-coding rRNA protein complexes are sequestered during times of stress—a step forward in the prebiotic chemical evolution by involving both coding and non-coding RNAs. This step is conspicuously missing from the origin of life lexicon in that it is never mentioned. PrPs localized to stress granules include the *S. Cerevisiae* protein kinase Sky1 prion-domain that regulates Np13, a nucleocytoplasmic mRNA shuttling protein for stress granules [77] and the human PrP TIA-1, that promotes assembly of stress granules and is involved in RNA binding [78]. If the functions of modern prion proteins reflect their ancient roles, then it seems that one of the important functions of ancient PrPs would have been both to sequester and to preserve a precious resource: RNA.

## 11. Prions at the Dawn of Life—A Summary

It is proposed that prion proteins are relics of an ancient prebiotic chemistry; and it is also proposed that these polymers were foundational for the chemical evolution of life on Earth. Most likely such polymers were synthesized initially from the thirteen α-amino acids listed in Table 1. Collectively, these “primordial” amino acids manifest a range of properties: six have hydrophobic aliphatic residues; two are hydrophilic and acidic; and two are polar, uncharged, and contain a side chain resulting in an interesting chemistry (Figure 1 upper panel). Specifically given the hydrophobic nature of these α-amino acids, this collection of amino acids would be expected to yield prions with a wide (albeit rudimentary) range of potential activities.

Although scientists do not yet possess a means by which to prove life’s origins, nevertheless, there is considerable circumstantial evidence from contemporary biology and chemistry to support a prion first hypothesis. In this paper we have discussed how prions and PrPs can act as carriers of information, as catalysts, as protectors for RNA and as supporters for metabolic processes, as well as early homeostatic binary switches. We have discussed the potential of prion proteins both for the possibility of shape mutation and for template-directed self-propagation of shape. We have also highlighted the resilience of prions and PrPs on exposure to the harsh conditions which were likely to have been present on early Earth. With this in mind, it could be construed that there is a high probability that prions could have been the priori molecules that instigated prebiotic chemistry towards the emergence of life on this planet.

## Figures and Tables

**Table 1:** Showing a list of all twenty α-amino acids used by all biology on the Earth for making proteins. (**a**) Trifonov’s (2004), sequential listing of reconstruction of appearance of amino acids as per evolutionary time-frame [10]. Trifonov’s list is mainly compared against that of Cobb’s and Bartlett’s comprehensive listing in (**g**) and (**h**), respectively. (**b**) Short properties of α-amino acid. (**c**) Reassessment of Miller’s 1953 electric discharge results [7] by Johnson et al. 2008 [9]. (**d**) Maury (2012) reported the first molecular replicator on the primitive Earth to be an informational amyloid: Glu-Gly-Gly-Ser-Val-Val-Ala-Ala-Asp [11]. (**e**) Ikehara (2005) generated ten α-amino acids via the proposed “GNC-SNS primitive genetic code hypothesis” (see Table 2). From these ten amino acids, he formulated the protein first hypothesis: GADV hypothesis [12]. (**f**) Gulik (2009) reported the first protein to be made up of prebiotic molecules as follows: DAKVGDGD = Asp-Ala-Lys-Val-Gly-Asp-Gly-Asp [13]. (**g**) Cobb’s (2014) reported α-amino acids inventory from chondrites [14]. (Although it is often thought that Tyr to be of biogenic origin but in 2009 Pizzarello and Holmes reported the presence of Tyr in CM2 and CR2 chondrites) [15]. (**h**) Data extracted from Bartlett’s (2002) paper shows amino acids’ relevance in catalytic propensity of Asp > Glu > Lys > Ser > Thr > Gly > Leu > Pro > Ile > Ala > Val (excluding Tyr and Phe) which may also be applicable to extra-terrestrial molecules [16]. (**i**) Cornell (2019) showed prebiotic α-amino acids binding to prebiotic fatty acid membranes in the presence of salt and Mg^2+^ [17]. Note: the first 9 α-amino acids (Gly to Thr) have a high tendency for formation of α-helices due to their hydrophobic side chains, especially in intramembrane environments [18].

(a) Trifonov 2004	(b) α-Amino Acid Properties	(c) Johnson (2008) Electric Discharge (Miller 1953)	(d) Maury (2012) First Replicator	(e) Ikehara 2005 GADV Hypothesis	(f) Gulik 2009 DAKVGDGDfirst Prebiotic Protein	(g) Cobb 2014 Meteorite Inventory	(h) Bartlett Catalytic Propensity of Residues	(i) Cornell (2019), Prebiotic Amino Acids/Fatty Membranes
(1) Gly	“Spacer” aliphatic residues	✔	✔	✔	✔	✔	✔	✔
(2) Ala	Hydrophobic aliphatic residues	✔	✔	✔	✔	✔	✔	✔
(3) Asp	Hydrophilic Acidic	✔	✔	✔	✔	✔	✔	
(4) Val	Hydrophobic aliphatic residues	✔	✔	✔	✔	✔	✔	✔
(5) Pro	Hydrophobic aliphatic residues			✔		✔	✔	
(6) Ser	Polar, uncharged, side chain	✔	✔			✔	✔	✔
(7) Glu	Hydrophilic Acidic	✔	✔	✔		✔	✔	
(8) Leu	Hydrophobic aliphatic residues			✔		✔	✔	✔
(9) Thr	Polar, uncharged, side chain					✔	✔	✔
(10) Arg				✔				
(11) Ile	Hydrophobic aliphatic residues					✔	✔	
(12) Gln				✔				
(13) Asn								
(14) His				✔				
(15) Lys	Basic, hydrophilic side chain				✔	✔	✔	
(16) Cys								
(17) Phe	Aromatic side chain-hydrophobic	✔				✔		
(18) Tyr	Aromatic side chain-hydrophilic					Pizzarello and Holmes (2009)		✔
(19) Met								
(20) Trp								

**Table 2:** Illustrates how the codons for 10 amino acids came to be as predicted by the GNC-SNS primitive genetic code hypothesis, where N and S represent either of four bases (A, U, G, and C) and G or C, respectively. The hypothesis contends that the universal genetic code originated from the GNC primordial code (four codons and four amino acids) through to the SNS primitive code (16 codons and 10 amino acids), culminating into his GADV hypothesis [12].

	U	C	A	G	
C	Leu (CUC)	Pro (CCC)	His (CAC)	Arg (CGC)	C
	Leu (CUG)	Pro (CCG)	Gln (CAG)	Arg (CGG)	G
G	Val (GUC)	Ala (GCC)	Asp (GAC)	Gly (GGC)	C
	Val (GUG)	Ala (GCG)	Glu (GAG)	Gly (GGG)	G

**Table 3:** Shows catalytic triads and examples of enzymes.

	Catalytic Triads	Examples of Enzymes
1	Ser-Glu-Asp	seldolisin proteases
2	Thr-Lys-Asp	Hydrolase L-asparaginase
3	Asp-Tyr-Lys	Aldo-keto reductase
4	Thr-His-His	Hydrolase phosphotransferase
5	Cys-His-Asp	cysteine proteases

## Data Availability

Not applicable.
